# Modeling *Culicoides* abundance in mainland France: implications for surveillance

**DOI:** 10.1186/s13071-019-3642-1

**Published:** 2019-08-06

**Authors:** Pierre Villard, Facundo Muñoz, Thomas Balenghien, Thierry Baldet, Renaud Lancelot, Viviane Hénaux

**Affiliations:** 1 CIRAD, UMR ASTRE, 34398 Montpellier, France; 2 ASTRE, CIRAD, INRA, Université de Montpellier, Montpellier, France; 3 Unité Epidémiologie et Appui à la Surveillance, Laboratoire de Lyon, Université de Lyon - ANSES, 31 Avenue Tony Garnier, 69007 Lyon, France; 4 Unité Microbiologie, Immunologie et Maladies Contagieuses, Institut Agronomique et Vétérinaire Hassan II, 10100 Rabat, Morocco; 5 CIRAD, UMR ASTRE, 10101 Rabat, Morocco

**Keywords:** *Culicoides*, Abundance modeling, Vector-free period, Count model, Bluetongue, France

## Abstract

**Background:**

Biting midges of the genus *Culicoides* Latreille (Diptera: Ceratopogonidae) are involved in the transmission of several viruses affecting humans and livestock, particularly bluetongue (BTV). Over the last decade, *Culicoides* surveillance has been conducted discontinuously and at various temporal and spatial scales in mainland France following the BTV epizootics in 2008–2009 and its reemergence and continuous circulation since 2015. The ability to predict seasonal dynamics and spatial abundance of *Culicoides* spp. is a key element in identifying periods and areas at high risk of transmission in order to strengthen surveillance for early detection and to establish seasonally disease-free zones. The objective of this study was to model the abundance of *Culicoides* spp. using surveillance data.

**Methods:**

A mixed-effect Poisson model, adjusted for overdispersion and taking into account temperature data at each trap location, was used to model the weekly relative abundance of *Culicoides* spp. over a year in 24 vector zones, based on surveillance data collected during 2009–2012. Vector zones are the spatial units used for *Culicoides* surveillance since 2016 in mainland France.

**Results:**

The curves of the predicted annual abundance of *Culicoides* spp. in vector zones showed three different shapes: unimodal, bimodal or plateau, reflecting the temporal variability of the observed counts between zones. For each vector zone, the model enabled to identify periods of vector activity ranging from 25 to 51 weeks.

**Conclusions:**

Although the data were collected for surveillance purposes, our modeling approach integrating vector data with daily temperatures, which are known to be major drivers of *Culicoides* spp. activity, provided areas-specific predictions of *Culicoides* spp. abundance. Our findings provide decisions makers with essential information to identify risk periods in each vector zone and guide the allocation of resources for surveillance and control. Knowledge of *Culicoides* spp. dynamics is also of primary importance for modeling the risk of establishment and spread of midge-borne diseases in mainland France.

**Electronic supplementary material:**

The online version of this article (10.1186/s13071-019-3642-1) contains supplementary material, which is available to authorized users.

## Background

Biting midges of the genus *Culicoides* Latreille (Diptera: Ceratopogonidae) are involved in the transmission of several viruses affecting both animals [e.g. bluetongue (BTV), Schmallenberg (SBV), Akabane, African horse sickness and epizootic hemorrhagic disease viruses] and humans (e.g. Oropouche fever virus). In Europe, the incursion of BTV and SBV in the last decade has caused substantial economic losses to farmers [1–3]. Since the large scale SBV epidemic that affected 29 European countries in 2011–2013, this disease appears to have settled to a low-level endemic circulation [4, 5] and is now recognized as a farm disease. In contrast, several European countries have been repeatedly affected by the circulation of both established and newly introduced BTV strains [6, 7]. Bluetongue (BT) is a disease regulated at the European level and, since 2000, the European Commission has established a series of regulations for control and surveillance in infected countries [8]. Control measures include vector control, restriction to movements of live ruminants from infected to non-infected regions and vaccination. Movement restrictions, which impose major technical and economic constraints to farmers, may be lifted in areas where evidence shows no virus circulation in livestock during vector-free periods. This decision requires a good knowledge of the temporal and spatial phenology of vector species.

In France, the main Mediterranean BTV vector, *Culicoides imicola* Keiffer, was detected for the first time on the island of Corsica in October 2000, just before the occurrence of important outbreaks of BTV serotype 2 (BTV-2) in the autumns of 2000 and 2001 [9, 10]. As a consequence, *Culicoides* surveillance was first implemented in Corsica and along the Mediterranean coast of the French mainland. This entomological surveillance was extended to the whole French mainland in 2009 to monitor vector activity following the introduction and spread of BTV-8 throughout the country in 2007–2008 [11]. The national-scale surveillance program ceased in 2012 and was implemented again in 2016–2018 following the re-emergence of BTV-8 in France in 2015 [12]. Currently, two BTV strains circulate in the French mainland (serotypes 4 and 8) and Corsica has a regulated status against several BTV strains (serotypes 1, 2, 4, 8 and 16) [13].

Entomological surveillance has been conducted by the French Agricultural Research Centre for International Development (CIRAD), mandated by The French Ministry of Agriculture and Food. During 2009–2012, *Culicoides* spp. surveillance covered the whole of mainland France with over 200 traps operating weekly or monthly depending on the season [11, 14]. During 2016–2018, the *Culicoides* spp. surveillance network has been optimized and operated in 24 zones, with one night of trapping per week at one site per zone from autumn to spring. These zones, recently named vector zones, were defined by an analysis (ascending hierarchical classification) of catch data collected during 2009–2012 to be homogeneous in terms of *Culicoides* species diversity and phenology (start and end of activity period). This entomological surveillance (which was active from November to next April) enabled the determination of periods without *Culicoides* vectors in each zone. The information provided by this network, coupled with the surveillance of viral circulation in livestock, allowed several French departments to be reported as BTV seasonally-free zones during the winters of 2016–2017 and 2017–2018, and thus restrictions on movements could be lifted for susceptible livestock from those zones. This status is critically important for livestock stakeholders to access the trade market (with no additional cost of serological and virological testing before the movement).

Our ability to predict the seasonal dynamics and spatial abundance of *Culicoides* spp. is a key element in determining high-risk transmission periods and areas to reinforce surveillance for early detection and to establish seasonally disease-free zones [8, 15]. This knowledge is also essential for modeling the transmission and spread of *Culicoides*-borne diseases and for identifying the most effective control measures [16, 17]. The objective of our study was to model the seasonal dynamics of *Culicoides* spp. in France using a combination of temperature and catch data collected during 2009–2012 for each vector zone. The results were compared with those predicted for two alternative spatial units: mainland France and iso-hygro-thermal zones, to demonstrate the relevance of vector zones as a spatial reference unit for surveillance and modeling of diseases transmitted by *Culicoides* spp.

## Methods

### Data

We used *Culicoides* spp. catch data obtained from 203 capture sites throughout mainland France between the second week of 2009 and the last week of 2012. This dataset includes information on the location of capture sites (latitude and longitude), the week of capture (trapping systematically occurs on Monday or Tuesday nights) and the number of specimens collected from each trap. *Culicoides* midges were collected with suction light traps (12 V, 8 W; manufactured by Onderstepoort Veterinary Institute, Pretoria, South Africa) installed from sunset to sunrise outside at 1.5–2.0 m above ground level immediately next to the stable or on a tree within < 30 m of the stable in close proximity to livestock. Traps were placed outdoors at exactly the same trapping location at the different sites throughout the entire study and operated one night on a monthly basis in winter and summer and on a weekly basis in spring and autumn. The samples were sent to CIRAD, the Interdepartmental Public Agency for Mosquito Control on the Mediterranean coast (EID-Med) or the Institute of Parasitology and Tropical Diseases of Strasbourg (IPPTS) for *Culicoides* species identification at the species level using relevant morphological identification keys [18, 19] and individual counting. We used data at the genus level, i.e. *Culicoides* spp. The catch data corresponds to the relative abundance (hereafter referred to as abundance) because only a fraction of the vector population is captured by the traps. Since the collections were performed in a standard manner, the numbers can be used to compare data between locations or sampling dates [20].

Weekly minimum and maximum air temperatures at an altitude of 2 m (in °C) were obtained for 2009–2012 from Meteo-France (available at https://donneespubliques.meteofrance.fr/). It provided data on an 8 km square lattice and we assigned the closest meteorological data to each capture site.

### Model

*Culicoides* and temperature data for each capture site were associated with the corresponding zone. The catch data consisted of the total number of captured *Culicoides* $Y_{ijl}$ from the capture site $i$ on year $j$ in week $l$. We modelled *Culicoides* counts for each vector zone with a Poisson model, adjusted for overdispersion, which included a spline [21] on the week number to account for seasonal variation in count, minimum air temperatures and difference between maximum and minimum air temperatures (which were centered and reduced), and random effects on both year and capture sites:

1 $$P\left( {Y_{ijl} = k} \right) \sim {\mathcal{P}}{\text{oisson}}\left( {\lambda_{ijl} } \right)$$

2 $$\log \left( {\lambda_{ijl} } \right) = \left( {\beta_{0} + u_{oi} + u_{oj} } \right) + \beta_{1} \times X_{l} + \beta_{2} \times \theta min_{ijl} + \beta_{3} \times \theta delta_{ijl}$$

where $Y_{ijl}$ is the number of *Culicoides* at site $i$ on year $j$ in week $l$; $\lambda_{ijl}$ is the rate parameter at site $i$ on year $j$ in week $l$; $X_{l}$ is the natural spline value for the week $l$; $\theta min_{ijl}$ is the minimum air temperature at site $i$ on year $j$ in week $l$; $\theta delta_{ijl}$ is the difference between maximum and minimum air temperature at site $i$ on year $j$ in week $l$; $\beta_{0}$ is the global intercept; $\beta_{1}$ is the slope for variable $X_{l}$; $\beta_{2}$ is the slope for variable $\theta min$; $\beta_{3}$ is the slope for variable $\theta delta$; and $u_{oi}$, $u_{oj}$ is the random effects of the site and the year on the intercept.

We used a natural spline with five degrees of freedom (*df*), which allowed one or two peaks in the *Culicoides* seasonal dynamics. In spatial units where the model did not converge, we reduced the *df* by a decrement of 1 *df* until the model finally converged.

The ability of the model to predict *Culicoides* abundance was estimated using the mean absolute error (MAE) and the root-mean-square error (RMSE). We calculated both indicators on the direct predictions to estimate the explanatory ability of the model variables and then by a cross-validation procedure to test the predictive ability of the model. For the cross-validation, we randomly partitioned the data into two sets of 90% for training and 10% for testing and calculated RMSE and MAE on the testing data. This process was performed 1000 times for each vector zone. Statistical analyses and graphical representations were performed using R with packages *splines* and *maptools* [22].

In order to evaluate the relevance of the vector zones as the reference partitioning for *Culicoides* surveillance, we tested the model introduced above on two alternative partitionings: no partitioning (i.e. mainland France considered as a unique spatial zone), and an iso-hygro-thermal partitioning. The comparison of model predictions among partitionings was based on two criteria. The first was the ability of the model to correctly predict the presence or absence of *Culicoides* for each week (estimated using a receiver operating characteristic (ROC) curve approach [23–25]). The second was the ability of the model to provide a realistic estimate of *Culicoides* abundance (estimated by the proportion of observed data within the confidence interval predicted by each model). The methods describing the development of the iso-hygro-thermal partitioning and the results of the comparison of model predictions among partitionings are described in Additional file 1: Text S1.

We produced annual curves of abundance for each vector zone using weekly averaged temperatures over the four studied years (2009–2012). For each vector zone, the beginning and the end of the seasonally *Culicoides*-free period were defined assuming a threshold of an estimated abundance of ten *Culicoides*, which indicates significant activity [26]. The cumulated abundance of *Culicoides* over one year was obtained by calculating the area under the predicted abundance curve, with the R package *pROC* [27]. For the ease of understanding, the cumulated abundance was then transformed to a mean weekly abundance.

Statistical analyses and graphical representations were performed using R [28] with the R package *tis* [29].

## Results

Each vector zone had on average 8.3 capture sites (median: 7.0; interquartile range: 5.0–11.0) during 2009–2012.

Model goodness-of-fit values and cross-validation results for each vector zone are provided in Additional file 2: Table S1. We note that the predicted values for the *Culicoides* abundance are very close to the observed values, except in four zones (1-3, 3-1, 3-3, 3-6) where extreme observed abundance resulted in large residuals and mathematically increased the MAE and RMSE values.

The mean effects and 95% confidence interval (CI) of the temperature variables (minimum temperature and temperature delta) estimated by the Poisson model for each zone are provided in Table 1. For five zones (in north-western France: 4-3, 4-5, 4-6; and eastern France: 1-2, 3-2), the overall effect of temperature was positive; for ten zones spread in the southern two-thirds of France (1-1, 1-4, 1-6, 1-7, 2-2, 2-3, 3-1, 3-3, 3-5, 5-5) the overall effect was negative; and in nine zones (1-3, 1-5, 1-8, 2-8, 3-4, 3-6, 3-8, 4-4, 6-8) the two temperature variables (minimum and delta) were found to have non-significant effects; by overall effect, we mean that effects are either both significant or one significant and the other non-significant.

**Table 1:** Effects of minimum temperature and temperature delta on *Culicoides* relative abundance (mean and 95% confidence interval, CI) estimated from the Poisson regression model for each vector zone in mainland France

Zone	Minimum temperature		Delta temperature	
	Coefficient (95% CI)	*P*-value	Coefficient (95% CI)	*P*-value
1-1	1.02 (0.91–1.15)	7.12 × 10^−1^	0.84 (0.77–0.93)	5.33 × 10^−4^*
1-2	1.13 (1.07–1.20)	7.92 × 10^−6^	0.99 (0.93–1.06)	7.78 × 10^−1^
1-3	1.05 (0.94–1.18)	3.86 × 10^−1^	1.08 (0.95–1.24)	2.28 × 10^−1^
1-4	0.86 (0.77–0.96)	6.51 × 10^−3^*	0.44 (0.31–0.62)	1.45 × 10^−5^*
1-5	1.02 (0.93–1.11)	7.17 × 10^−1^	0.99 (0.90–1.08)	7.76 × 10^−1^
1-6	0.83 (0.76–0.90)	1.85 × 10^−5^*	0.98 (0.91–1.05)	5.59 × 10^−1^
1-7	0.85 (0.72–1.00)	4.75 × 10^−2^*	1.16 (1.00–1.35)	5.23 × 10^−2^
1-8	0.98 (0.89–1.09)	7.42 × 10^−1^	0.91 (0.80–1.03)	1.39 × 10^−1^
2-2	0.91 (0.87–0.94)	2.44 × 10^−7^*	1.02 (0.97–1.07)	5.05 × 10^−1^
2-3	1.01 (0.97–1.05)	7.17 × 10^−1^	0.91 (0.87–0.94)	3.78 × 10^−6^*
2-8	1.06 (0.95–1.19)	2.91 × 10^−1^	1.02 (0.91–1.15)	7.43 × 10^−1^
3-1	0.99 (0.90–1.09)	8.35 × 10^−1^	0.86 (0.79–0.93)	2.67 × 10^−4^*
3-2	0.99 (0.93–1.05)	6.69 × 10^−1^	1.08 (1.01–1.17)	3.74 × 10^−2^*
3-3	0.93 (0.87–1.00)	3.80 × 10^−2^*	1.03 (0.96–1.11)	3.74 × 10^−1^
3-4	1.06 (0.96–1.16)	2.63 × 10^−1^	1.04 (0.92–1.17)	5.39 × 10^−1^
3-5	0.84 (0.78–0.91)	3.42 × 10^−6^*	1.01 (0.93–1.10)	8.30 × 10^−1^
3-6	0.95 (0.87–1.04)	2.50 × 10^−1^	0.95 (0.87–1.04)	2.62 × 10^−1^
3-8	0.99 (0.88–1.10)	8.04 × 10^−1^	1.05 (0.93–1.18)	4.34 × 10^−1^
4-3	1.20 (1.09–1.31)	1.38 × 10^−4^*	1.02 (0.91–1.15)	7.47 × 10^−1^
4-4	1.10 (0.98–1.24)	1.17 × 10^−1^	1.04 (0.911 1.18)	5.77 × 10^−1^
4-5	1.07 (1.00–1.15)	5.42 × 10^−2^	1.15 (1.06–1.25)	9.13 × 10^−4^*
4-6	1.12 (1.03–1.22)	1.14 × 10^−2^*	1.14 (1.03–1.25)	7.85 × 10^−3^*
5-5	0.88 (0.72–1.08)	2.14 × 10^−1^	0.72 (0.57–0.91)	8.25 × 10^−3^*
6-8	1.02 (0.92–1.13)	7.04 × 10^−1^	0.95 (0.85–1.06)	3.56 × 10^−1^

*Note*: Significant *P*-values are indicated by *

**Fig. 1:**
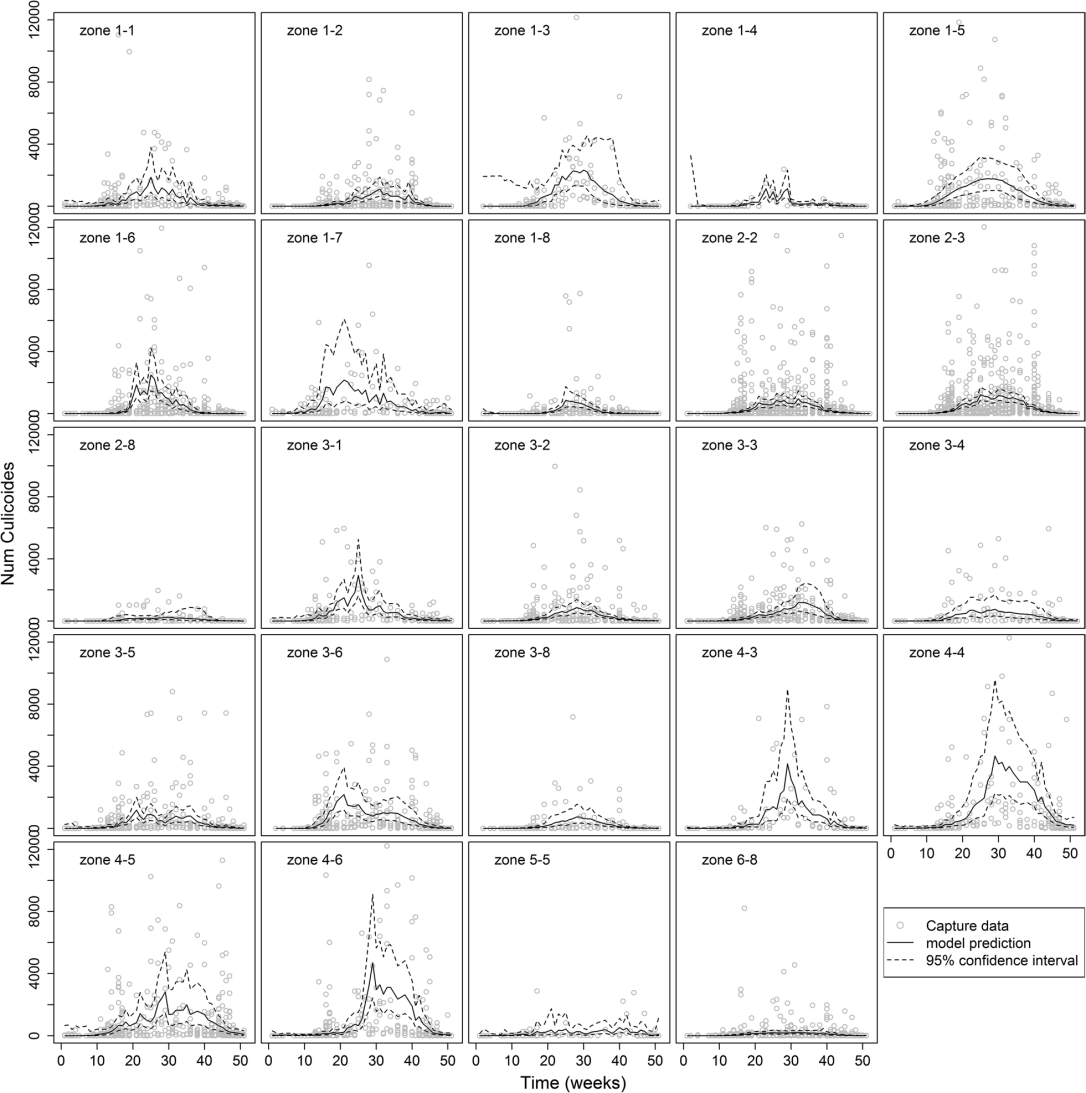
Model-predicted *Culicoides* abundance for each vector zone in mainland France based on 2009–2012 catch data. Dots represent capture data, solid line represents the model prediction, dashed lines represent 95% confidence interval

**Fig. 2:**
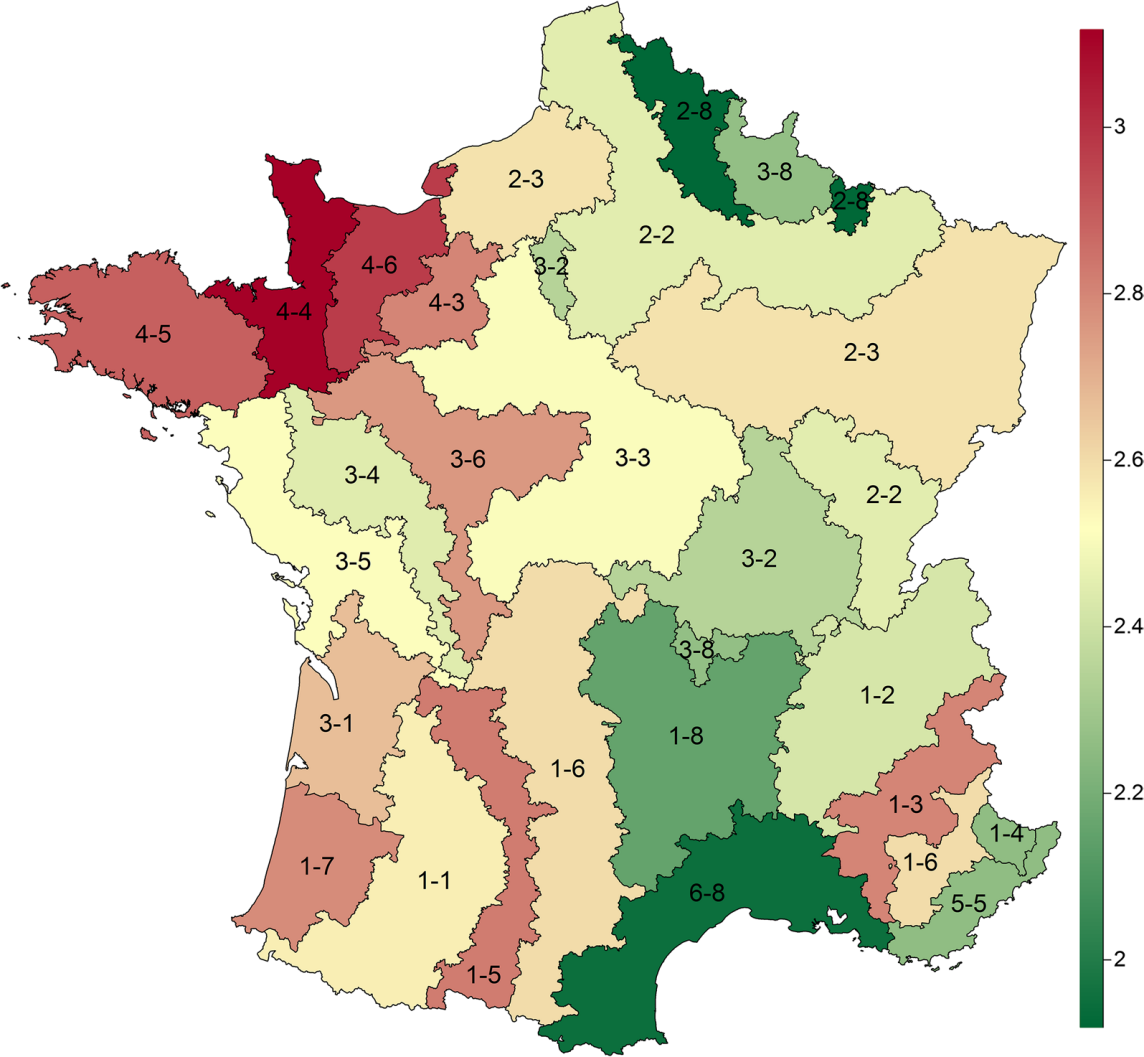
Weekly mean *Culicoides* abundance in each vector zone in mainland France on a logarithmic scale. Some vector zones are made of two non-contiguous areas

The curves of predicted annual *Culicoides* abundance in vector zones showed three alternative shapes (Fig 1): unimodal (e.g. zone 4-3), bimodal (e.g. zone 3-6) or plateau-like (e.g. zone 3-4), reflecting the temporal variability in observed counts among zones. Predicted maximum abundance varied also strongly among vector zones from about 200 *Culicoides* (zones 2-8 and 6-8) to over 4000 *Culicoides* at peak (zones 4-3, 4-4 and 4-6). The cumulative *Culicoides* abundance varied strongly among vector zones from about 80 to 1310 *Culicoides* on average per week (median: 344; interquartile range: 215–624; Table 2, Fig 2). Overall, the vector period lasted between 25 and 51 weeks, starting between weeks 1 (early January) and 15 (mid-April) and ending between weeks 43 (end of October) and 51 (mid-December) (Table 2).

**Table 2:** Predicted period of vector activity in each vector zone in mainland France assuming an abundance threshold of ten *Culicoides*

Zone	Vector period			Week of peak	Abundance at peak	Weekly mean abundance
	Starting week	Ending week	Duration			
1-1	8	51	44	25	1868	364
1-2	13	46	34	29	1104	267
1-3	13	49	37	30	2334	637
1-4	13	47	35	23; 29	1137; 1140	181
1-5	8	50	43	27	1778	674
1-6	14	43	30	25	2495	399
1-7	7	51	45	21	2168	620
1-8	17	41	25	25	860	144
2-2	11	49	39	32	964	282
2-3	10	49	40	25	1218	389
2-8	15	45	31	29	242	83
3-1	9	51	43	25	2936	472
3-2	11	49	39	28	857	224
3-3	12	47	36	32	1195	322
3-4	12	51	40	22; 29	693; 757	280
3-5	8	49	42	21	1186	325
3-6	11	49	39	21	2171	578
3-8	13	47	35	28	730	187
4-3	11	51	41	29	4167	635
4-4	7	51	39	29	4661	1309
4-5	7	51	45	29	2825	785
4-6	1	51	51	29	4712	953
5-5	8	51	44	25; 40	435; 527	183
6-8	12	47	36	25; 31	200; 208	89

## Discussion

In the present study, we modeled and quantified the weekly relative abundance of *Culicoides* spp. over a year in mainland France, using partitioning of the territory in vector zones and taking into account temporal and spatial variations in temperatures within those zones. Several studies have described the diversity and distribution of species in mainland France from surveillance data, yet, to our knowledge, our study is the first to provide zone-specific predictions of *Culicoides* abundance, which is critical for modeling the risk of establishment and spread of midge-borne diseases [30, 31] and implement risk-based surveillance and control measures.

The predicted curves of *Culicoides* abundance showed a strong seasonal pattern, reflecting the dependence of the *Culicoides* life-cycle on climatic conditions [32, 33], with poor tolerance of midges to low temperatures [34]. Indeed, during the cold season under temperate climates, most adult *Culicoides* disappear and the species survive as larvae (either due to true larval diapausing or to the prolonged duration of larval development at lower temperatures) [35]. Then, when temperatures start increasing, adults emerge and populations grow progressively to reach a peak of abundance in spring or summer depending on locations, as a function of spring temperatures and summer dryness. Indeed, temperature decreases the larval development time, the time between two blood meals, and therefore increases the laying frequency, which leads to a positive effect on the population dynamics (and its growth), and therefore we expected the temperature to have a positive effect on abundance [36]. Conversely, temperature is negatively correlated with survival [36]. Thus, there are temperature ranges for which the impact on abundance is positive, and others for which the impact on abundance is negative. It results in positive correlations in regions where temperatures do not reach high values, negative correlations in regions with high summer temperatures, or even non-linear effects. Overall, our results underlined marked differences in the shape and level of the abundance curves (with bimodal, unimodal or plateau-like patterns) among vector zones. These temporal and spatial differences reflect the large diversity of *Culicoides* species in mainland France, which is caused by the variety of climatic conditions, edaphic factors and farming practices. The subgenus *Avaritia* Fox (composed primarily of the *Culicoides obsoletus* (Meigen)/*Culicoides scoticus* Downes & Kettle complex, *C. imicola*, *Culicoides dewulfi* Goetghebuer and *Culicoides chiopterus* (Meigen)) is the most prevalent, representing more than 80% of captures, followed by the subgenus *Culicoides* (primarily, *Culicoides newsteadi* Austen and *Culicoides pulicaris* (Linnaeus)) . While *C. imicola* and *C. newsteadi* are common along the Mediterranean coast and in Corsica, other species are more widespread in temperate areas, with variation in abundance between oceanic, continental or mountain regions [14]. The dominant *C. obsoletus/C. scoticus* exhibits bimodal patterns of abundance in southern regions of France (with peaks in late spring and fall as populations decrease during summer due to dryness), while unimodal patterns (with a peak in summer) are more frequent the north of the country [11, 14]. Indeed, although temperatures are known as a major driver of *Culicoides* larvae development and adult activity, other variables (including rainfall, humidity, soil texture, normalized difference vegetation index, elevation, farming systems, densities of wild vertebrate hosts and land cover) may influence the phenology, distribution and abundance of midge species.

In order to simplify and reduce the cost of the monitoring of midge populations, entomological surveillance in France has relied, over the last years, on a spatial partitioning of the territory, defined from an ascending hierarchical classification of historical (2009–2011) *Culicoides* records. The comparison of model predictions based on this vector-based partitioning to those obtained with no partitioning (Additional file 1: Text S1, Figures S3, S5, S6) underlined the importance of modeling *Culicoides* abundance at a local scale to account for the spatial variation in both the distribution of species and the seasonal dynamics. Furthermore, our study showed that the vector-based partitioning provided a similar or better fit to catch data than an iso-hygro-thermal partitioning (Additional file 1: Text S1, Figures S1, S2, S4–S6), underlining the adequacy of the vector partitioning for planning surveillance and disease control activities.

The model included all available data on *Culicoides* collected during a four-year period (2009–2012), which allowed smoothing the effect of rare extreme or mild climatic events. However, we stress that the predicted vector abundance may be misjudged to some extent for different reasons. First, the data included zero counts. While some nil values may reflect the absence of vector, in other cases, zero counts may have resulted from adverse weather conditions on the day of trapping or technical problems with the trap. We decided to include all data in the model to capture the maximum variability even if zero counts were observed during the vector activity period. The use of a Poisson model adjusted for overdispersion allowed us to reduce the influence of the excess of zero counts on the estimation of abundance during the vector period. Secondly, among all *Culicoides* species recorded in France, only some have been connected with BTV transmission. *Culicoides imicola* and, to a lower extent, *C. newsteadi* are considered the main BTV vectors in the Mediterranean area, while *C. obsoletus*, *C. scoticus*, *C. dewulfi*, *C. chiopterus* and *C. pulicaris* (which are the most abundant and widely distributed species in mainland France) are involved as BTV vectors in other parts of Europe [37–46]. Virus isolations from field-collected *C. imicola* [47] and the reproduction of the transmission cycle in this species in experimental conditions [48] have proven this species to be a BTV vector. Likewise, *C. newsteadi*, *C. obsoletus*, *C. scoticus*, *C. dewulfi*, *C. chiopterus* and *C. pulicaris* (which are the most abundant and widely distributed species in mainland France) have either been found positive in field-collected samples [37–45, 49] or in experimentally-infected individuals [46] which suggests that they might act as vector species. These assertions are generally scientifically accepted [50] even if the vector competence of these species has not been comprehensively assessed in the laboratory due to technical issues, in particular the difficulties in feeding and maintaining *Culicoides*. As the species involved in the transmission of diseases are not exhaustively identified [37, 40, 43, 44, 46, 48, 51–57], we decided to use total *Culicoides* counts without distinction of species, which means that predicted weekly abundances may slightly overestimate the number of BTV vectors; however, the fact that species specified above represent almost 90% of all collected *Culicoides* in France makes us confident that the use of all *Culicoides* abundance data for risk assessments are valid. On the other hand, aggregating species might represent a problem for identifying accurate temporal and spatial patterns, as different species might exhibit different seasonal trends even in the same environment [58].

The spatial variation in abundance justifies the use of a regional policy for *Culicoides* surveillance and disease control. *Culicoides*-borne viruses like BTV and SBV cannot be transmitted to the susceptible host species in absence of adult vectors. Therefore, the European Union alleviates restriction measures during periods of vector inactivity, assuming that under the commonly used threshold of five parous females per trap per night, *Culicoides* populations are considered as inactive [8]. Our models did not include information about sex or age status of captured *Culicoides*; therefore, we decided to use a threshold of ten *Culicoides* per trap per night as a limit for declaring freedom of adult activity.

The fact that less than 5% of the total *Culicoides* collected using suction light traps are males suggests that not considering sex in our catch data does not affect the quality of our conclusions. Yet, the proportion of parous females in the *Culicoides* population may vary seasonally [59, 60]. These limits may alter the predictions of weekly abundance of *Culicoides* vectors and potentially overestimate the length of the activity period. We stress that the threshold of five parous females is conservative: it is likely that an abundance of more than five parous females/trap/night is required for BTV transmission to begin, but the exact threshold is not known [61, 62]. This evidence calls for more studies to refine this threshold, adjusted for the factors that may alter BTV transmission, such as vector longevity, biting rate and viral replication rate (which are highly dependent on the temperature) and disease prevalence in hosts.

Given the continuing need for optimizing the cost-effectiveness of animal disease surveillance, the knowledge of weekly *Culicoides* abundance in each zone creates new opportunities for a more efficient organization of field actors and allocation of resources for surveillance. Indeed, our study provides key input to conduct both serological and entomological surveillance during limited time windows before the predicted start and end of the vector in each zone. It could also be used to facilitate the planning of vector control strategies and increase their efficiency.

## Conclusions

Our study provides estimates of weekly abundance of *Culicoides* for 24 zones, defined to be homogenous in terms of vector diversity, inactivity period and species phenology, in mainland France. This study showed the relevance of the vector partitioning (based on 24 traps versus about 160 traps previously). Beyond the value of these results for allocating efficiently the surveillance effort and resources, the knowledge of local *Culicoides* abundance is an essential component of epidemiological models to simulate the risk of exposure of susceptible hosts to midge-borne diseases (e.g. [17]) and to identify the most appropriate measures for control.

## Additional files

**Additional file 1: Text S1.** *Culicoides* abundance using spatial units alternative to vector zones. **Figure S1.** Iso-hygro-thermal partitioning of mainland France. **Figure S2.** Distribution of minimum, maximum and average fortnight temperature and average specific humidity in each iso-hygro-thermal zone in mainland France. For each zone (cluster), the solid line represents the median value and dashed-lines the first and third quartiles of the distribution. **Figure S3.** Predicted *Culicoides* abundance in mainland France with no partitioning from the model. **Figure S4.** Predicted *Culicoides* abundance for each iso-hygro-thermal zone from the model in mainland France. **Figure S5.** ROC curves for the three spatial scales. **Figure S6.** Boxplot and distribution of the proportion of observed values within the predicted confidence interval for the three spatial scales.

**Additional file 2: Table S1.** Values (median and interquartile range) of the mean absolute error (MAE) and root mean square error (RMSE) for each vector zone.

## Data Availability

Data supporting the conclusions of this article are included within the article and its additional files. Capture data are available upon request from the French Ministry of Agriculture and Food. The outputs of the climate model can be found (for non-commercial usage) on the dedicated website: https://donneespubliques.meteofrance.fr/?fond=produit&id_produit=230&id_rubrique=40.

## Abbreviations

BTV: bluetongue virus
SBV: Schmallenberg virus
CIRAD: French Agricultural Research Centre for International Development
ANSES: French Agency for Food, Environmental and Occupational Health & Safety
EID-Med: Interdepartmental Public Agency for Mosquito Control on the Mediterranean coast
IPPTS: Institute of Parasitology and Tropical Diseases of Strasbourg
*df*: degrees of freedom
MAE: mean absolute error
RMSE: root-mean-square error
CI: confidence interval
