# Supplementary material for: Modeling Culicoides abundance in mainland France: implications for surveillance
Source: Parasit Vectors. 2019 Aug 6;12:391. doi: 10.1186/s13071-019-3642-1 (PMC6683357; doi:10.1186/s13071-019-3642-1)
Supplement: Supplementary file 1 — Additional file 1: Text S1. Culicoides abundance using spatial units alternative to vector zones. Figure S1. Iso-hygro-thermal partitioning of mainland France. Figure S2. Distribution of minimum, maximum and average fortnight temperature and average specific humidity in each iso-hygro-thermal zone in mainland France. For each zone (cluster), the solid line represents the median value and dashed-lines the first and third quartiles of the distribution. Figure S3. Predicted Culicoides abundance in mainland France with no partitioning from the model. Figure S4. Predicted Culicoides abundance for each iso-hygro-thermal zone from the model in mainland France. Figure S5. ROC curves for the three spatial scales. Figure S6. Boxplot and distribution of the proportion of observed values within the predicted confidence interval for the three spatial scales. [file 13071_2019_3642_MOESM1_ESM.docx]

**Additional file 1:** **Text S1.** *Culicoides* abundance using spatial units alternative to vector zones

# Materials and methods

The model predicting the *Culicoides* abundance (described in the main text) was conducted for two alternative spatial partitionings of the country: no partitioning (i.e., mainland France considered as a unique spatial zone) and an iso-hygro-thermal partitioning.

Statistical analyses and graphical representations were performed using R [1] with packages pROC [2].

## Iso-hygro-thermal partitioning

The iso-hygro-thermal partitioning of the territory aimed to group communes (a level of administrative division in France equivalent to that of the municipalities) similar in temperature and humidity that are geographically close. We obtained daily (minimum, maximum and average) values of two meters altitude temperature and daily average specific humidity on an 8 km square lattice for the 2009–2012 period, from Meteo-France (available on <https://donneespubliques.meteofrance.fr/>) [3, 4]. We computed the average fortnightly values of each meteorological variable across our four years for each commune. The communes were then grouped in two steps. First, we performed a multiple factorial analysis (MFA) (using the R package ade4 [5]) on four blocks of variables: three blocks of fortnightly temperature values (minimum, maximum and average temperatures) and one block of fortnightly relative humidity values. Secondly, we performed a hierarchical ascendant clustering [6] (package geoclust) and trimmed the hierarchical clustering tree to obtain a number of clusters close to the number of vector zones. We then performed a hierarchical ascendant clustering with spatial constraints (HACSC) [6] (package geoclust) using a Ward-like hierarchical clustering approach with two dissimilarity matrices: the first was built on the factorial coordinates of the communes derived from the MFA and the second on the distance between the communes’ centroids A mixing parameter α was used to set the importance of each dissimilarity matrix in the clustering procedure. A α value of 0 indicates that the hierarchical clustering is based solely on the meteorological matrix (derived from the MFA), a value of 1 indicates that the hierarchical clustering is based solely on the matrix of geographical distances, and a value of 0.5 indicates a similar weight of both matrices. With the number of clusters selected previously, we chose the α value which offered the best compromise between meteorological and geographical homogeneity, as described in [6]. Communes belonging to a cluster that were isolated within another cluster, i.e. surrounded by communes belonging to another cluster, were converted into this cluster.

## Comparison of results from the three modeling scales

Two criteria were used to compare predictions between the three models. The first consisted in the ability of the model to correctly predict the presence or absence of *Culicoides* for each week. We used a Receiver Operating Characteristic (ROC) curve approach [7-9] and calculated the area under the curve for the three spatial units. Under this method, the spatial unit giving the higher area under the curve would be considered the best for this criterion.

The second criterion was the ability of the model to provide a realistic estimate of *Culicoides* abundance. For each capture site, we computed the proportion of observed data within the confidence interval predicted by each model. For each modeling scale, we calculated the distribution of this indicator between capture sites.

# Results

## Iso-hygro-thermal partitioning

Based on a Ward dendrogram, we computed the inertia gain for each height of cut in the tree and the local maximum of the inertia gain correspond to 2, 8, 15, 18, 22 and 27 zones. We decided to choose a partitioning with 22 zones to be close to the number of zones in the vector partitioning (**Figure S1**). We selected an α of 0.5 because it offered the best compromise between meteorological homogeneity and compact zones. The allocation of 331 isolated communes (over 35,000, 0.95%) (i.e. surrounded by communes with another allocation) has been modified to be similar to neighboring communes. The distribution of meteorological data (weekly minimum, maximum and average temperatures and average specific humidity) for the 22 zones is summarized in **Figure S2** and highlights the meteorological homogeneity in each zone. Each iso-hygro-thermal zone had an average of 9.4 capture sites (median: 9.0; interquartile range [5.0, 12.0]) from 2009 to 2012.


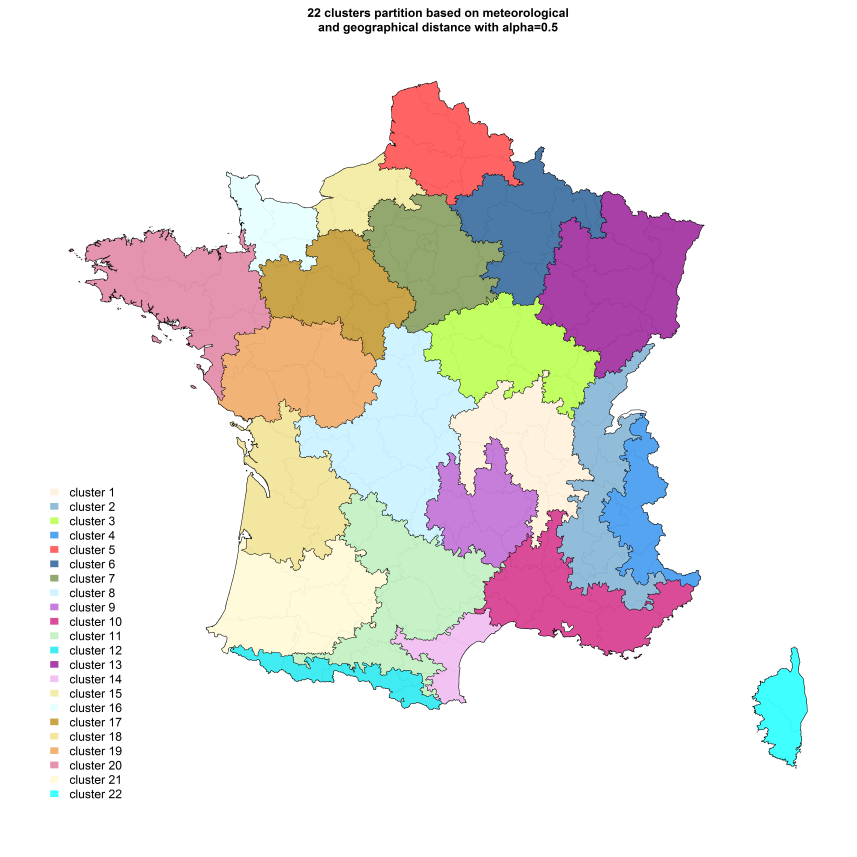


**Figure S1.** Iso-hygro-thermal partitioning of mainland France


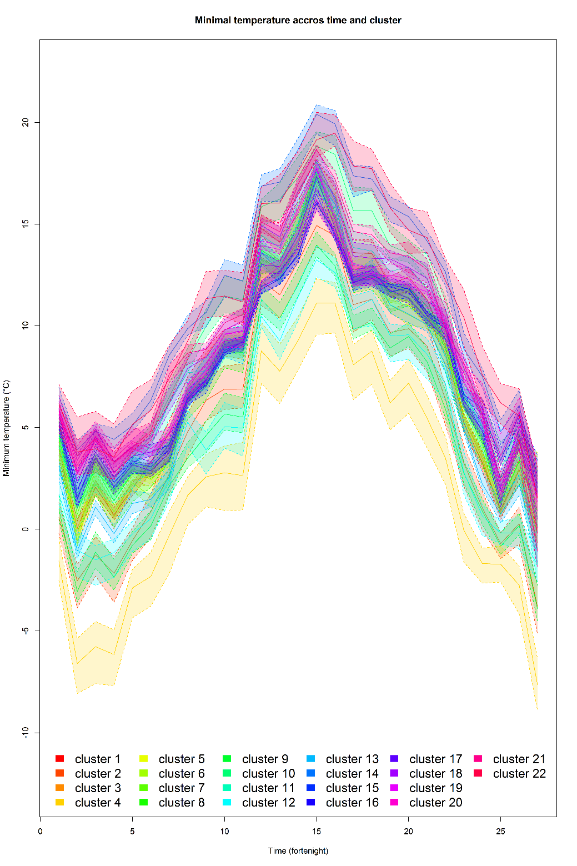

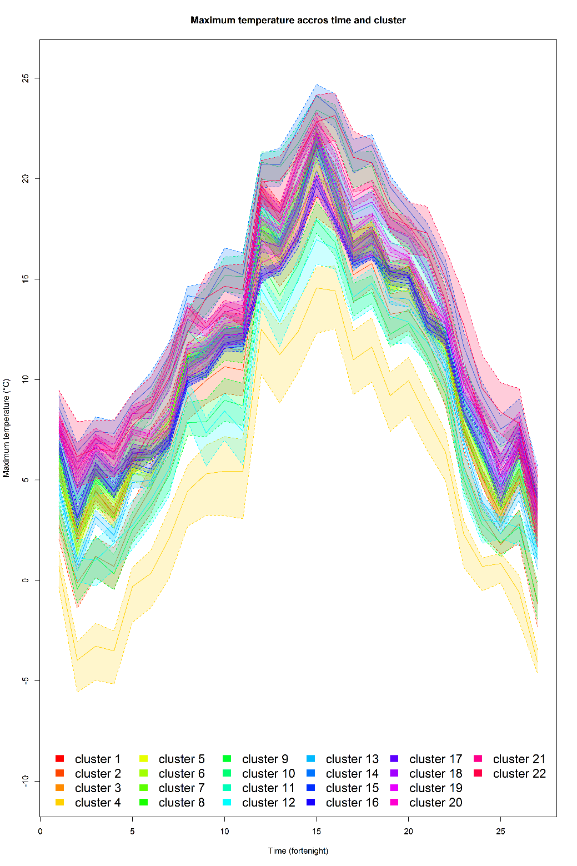

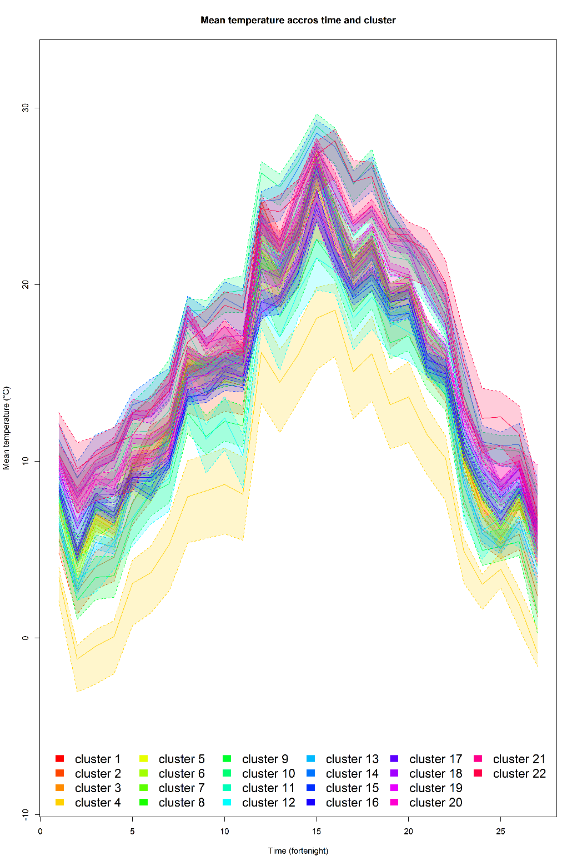

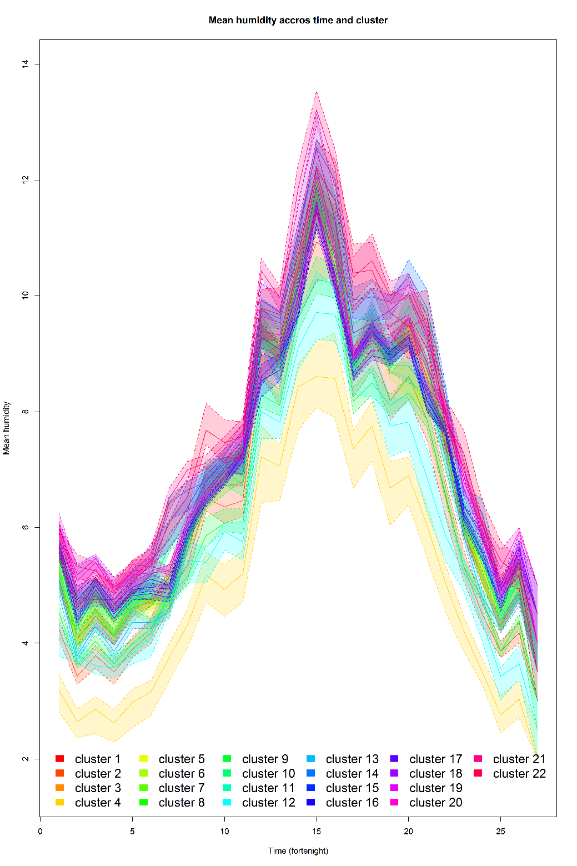


**Figure S2.** Distribution of minimum, maximum and average fortnight temperature and average specific humidity in each iso-hygro-thermal zone in mainland France. For each zone (cluster), the solid line represents the median value and dashed-lines the first and third quartiles of the distribution.

## Model predictions

The model applied in mainland France (i.e., without partitioning) predicted a unimodal curve with one peak around 1,150 *Culicoides* in week 29; the onset and end of the vector’s activity period were respectively weeks 10 and 49 (**Figure S3**). Model-based predictions of weekly *Culicoides* abundance in each iso-hygro-thermal zone showed unimodal, bimodal or plateau-like seasonal patterns (**Figure S4**). In some zones, the model was not even able to converge, which reduced the degree of freedom, so the values predicted for areas 12 and 15 are not available.


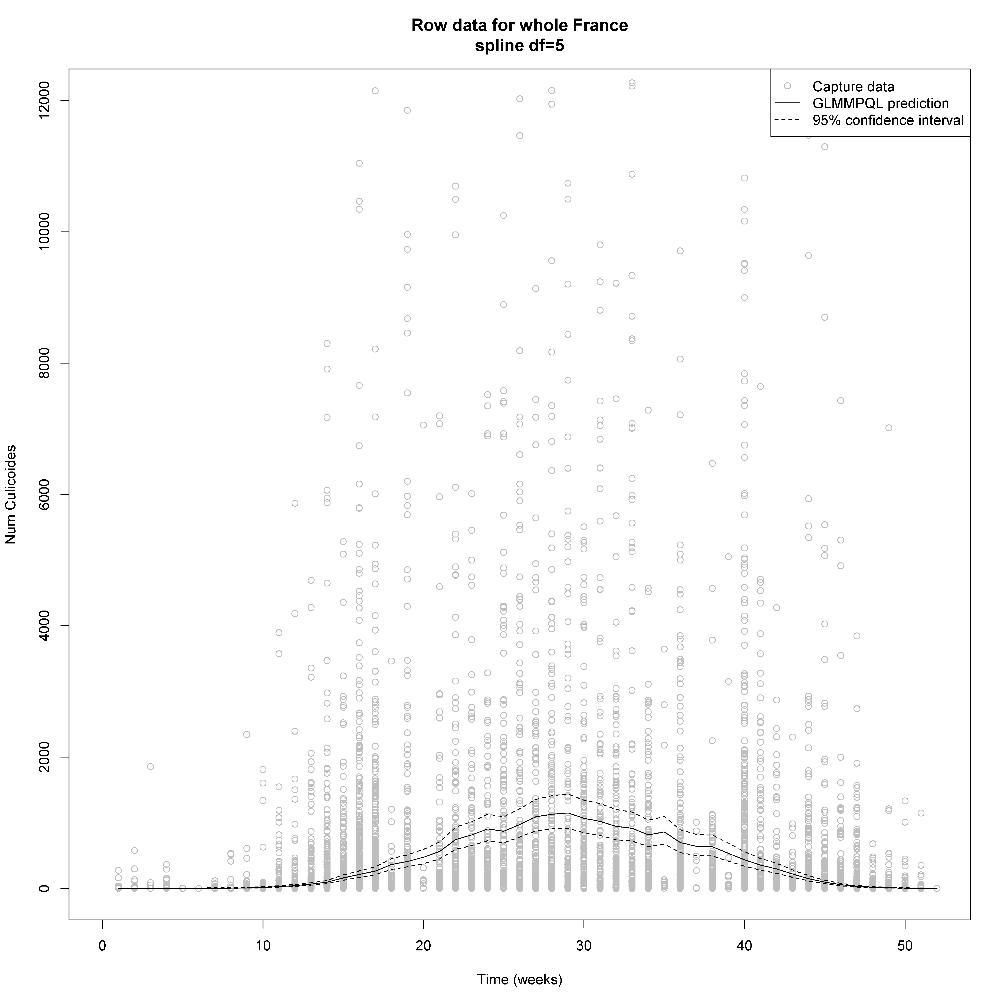


**Figure S3** Predicted *Culicoides* abundance in mainland France with no partitioning from the model


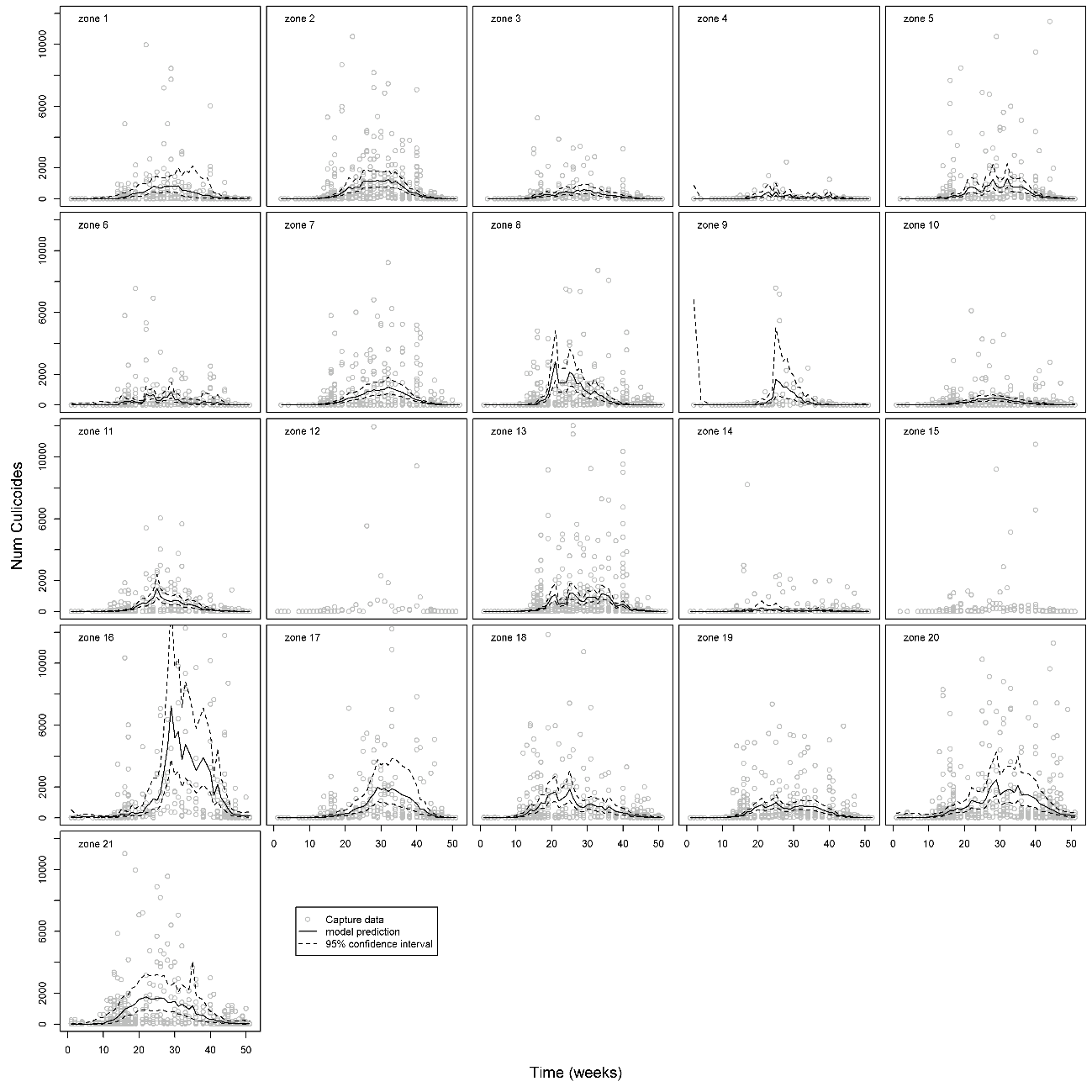


**Figure S4.** Predicted *Culicoides* abundance for each iso-hygro-thermal zone from the model in mainland France

## Comparison of model goodness-of-fit

The model based on the vector partitioning provided the highest ROC AUC (0.877 [95% confidence interval: 0.871-0.882]), followed by no partitioning model (0.859 [0.853-0.865]) and the iso-hygro-thermal partitioning model (0.821 [0.814-0.828]) (**Figure S5**).


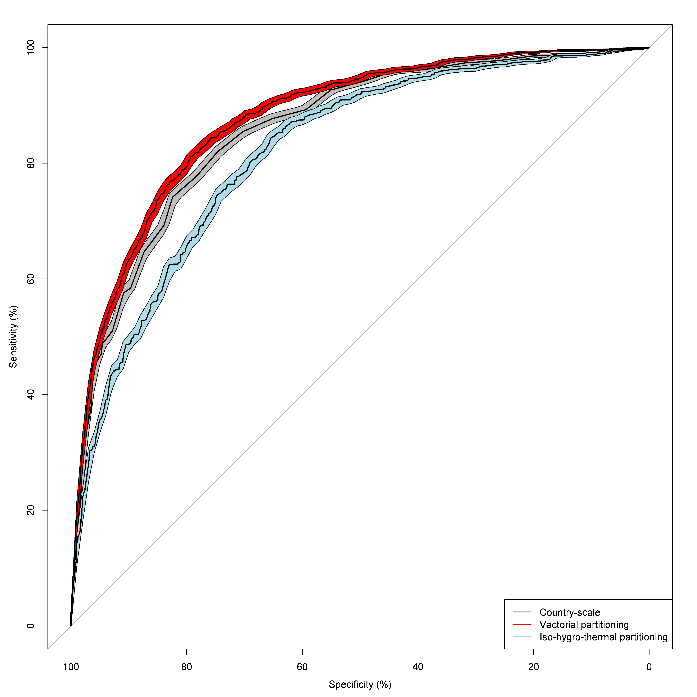


**Figure S5.** ROC curves for the three spatial scales

Models based on the vector or iso-hygro-thermal partitioning provided a similar distribution of the proportion of observed data within the predicted confidence interval, with less variability for vector partitioning. The model based on no partitioning predicted a realistic abundance in about 30% of weeks (**Figure S6**).


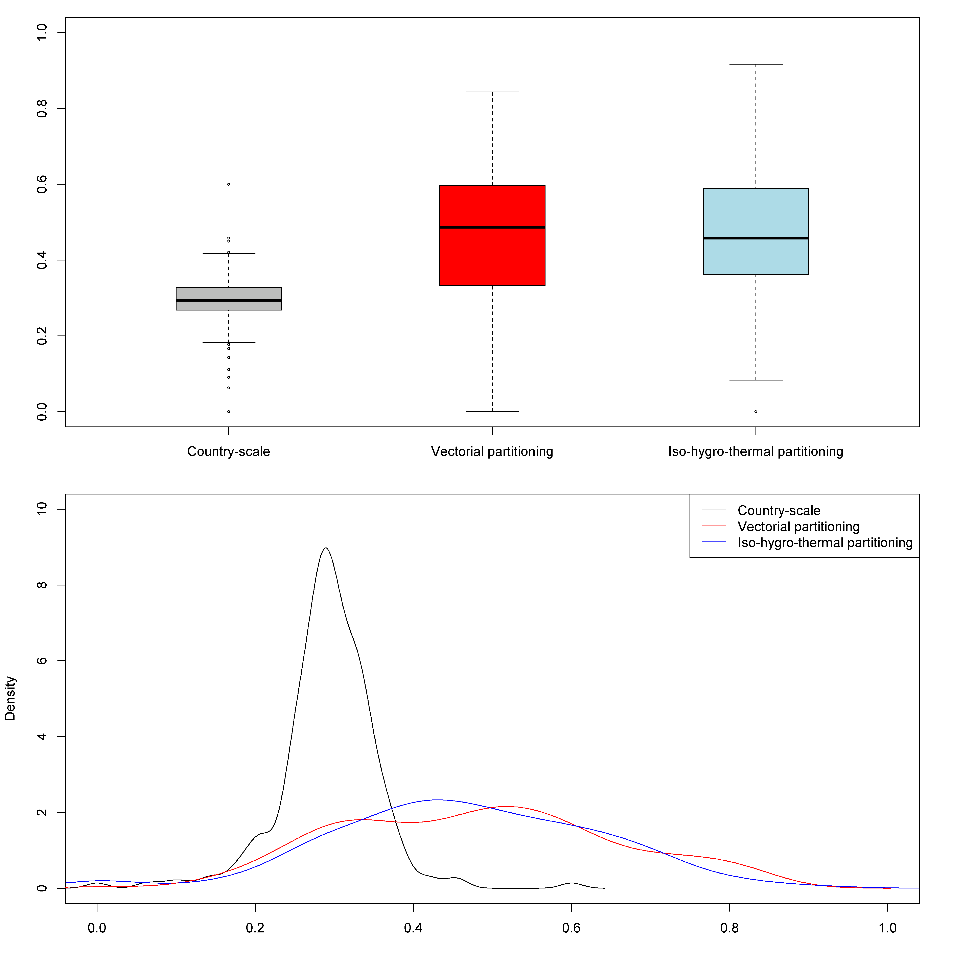


**Figure S6.** Boxplot and distribution of the proportion of observed values within the predicted confidence interval for the three spatial scales

# References

1. R Development Core Team. R: A language and environment for statistical computing. Vienna, Austria: R Foundation for Statistical Computing; 2015.

2. Robin X, Turck N, Hainard A, Tiberti N, Lisacek F, Sanchez J-C, et al. pROC: an open-source package for R and S+ to analyze and compare ROC curves. BMC Bioinform. 2011;12:77.

3. Le Moigne P. Description de l'analyse des champs de surface sur la France par le système SAFRAN. 2002.

4. Pagé C. Format des données SAFRAN et scénarios climatiques désagrégés au CERFACS. Centre Européen de Recherche et de Formation Avancée en Calcul Scientifique (CERFACS). 2008;Technical Report TR/CMGC/08/27.

5. Chessel D, Dufour AB, Thioulouse J. The ade4 package -I- One-table methods. R News. 2004;4:5-10.

6. Chavent M, Kuentz-Simonet V, Labenne A, Saracco J. ClustGeo: an R package for hierarchical clustering with spatial constraints. Comput Stat. 2018.

7. Griner PF, Mayewski RJ, Mushlin AI, Greenland P. Selection and interpretation of diagnostic tests and procedures. Principles and applications. Annals of Internal Medicine. 1981:557–92.

8. Metz CE. Basic principles of ROC analysis. Semin Nucl Med. 1978;8:283–98.

9. Zweig MH, Campbell G. Receiver-operating characteristic (ROC) plots: a fundamental evaluation tool in clinical medicine. Clin Chem. 1993;39:561.
