# Supplementary material for: Modeling Culicoides abundance in mainland France: implications for surveillance
Source: Parasit Vectors. 2019 Aug 6;12:391. doi: 10.1186/s13071-019-3642-1 (PMC6683357; doi:10.1186/s13071-019-3642-1)
Supplement: Supplementary file 2 — Additional file 2: Table S1. Values (median and interquartile range) of the mean absolute error (MAE) and root mean square error (RMSE) for each vector zone. [file 13071_2019_3642_MOESM2_ESM.docx]

**Additional file 2: Table S1.** Values (median and interquartile range) of the mean absolute error (MAE) and root mean square error (RMSE) for each vector zone.

| Zone | Direct predictions | | Cross-Validation | |
| --- | --- | --- | --- | --- |
|  | MAE | RMSE | MAE | RMSE |
| 1-1 | 8 | 77 | 311 [213 ; 458] | 706 [428 ; 1541] |
| 1-2 | 2 | 12 | 200 [159 ; 244] | 541 [413 ; 701] |
| 1-3 | 369 | 6,613 | 330 [242 ; 448] | 727 [529 ; 1131] |
| 1-4 | 2 | 5 | 65 [37 ; 153] | 113 [74 ; 348] |
| 1-5 | 4 | 42 | 615 [500 ; 741] | 1285 [1019 ; 1682] |
| 1-6 | 7 | 115 | 499 [297 ; 897] | 2189 [1048 ; 5801] |
| 1-7 | 7 | 78 | 927 [593 ; 1430] | 2055 [1181 ; 4194] |
| 1-8 | 2 | 10 | 142 [84 ; 233] | 585 [254 ; 1087] |
| 2-2 | 1 | 2 | 398 [328 ; 494] | 1390 [1011 ; 1964] |
| 2-3 | 1 | 2 | 352 [302 ; 405] | 1083 [858 ; 1320] |
| 2-8 | 1 | 3 | 108 [86 ; 132] | 289 [223 ; 338] |
| 3-1 | 19 | 157 | 353 [270 ; 467] | 753 [523 ; 1018] |
| 3-2 | 1 | 2 | 216 [169 ; 271] | 635 [456 ; 847] |
| 3-3 | 369 | 8,257 | 273 [204 ; 381] | 756 [540 ; 1731] |
| 3-4 | 78 | 979 | 293 [210 ; 395] | 643 [471 ; 955] |
| 3-5 | 1 | 4 | 331 [261 ; 418] | 912 [679 ; 1163] |
| 3-6 | 689 | 13,747 | 561 [448 ; 725] | 1475 [985 ; 1943] |
| 3-8 | 1 | 2 | 148 [99 ; 210] | 383 [228 ; 576] |
| 4-3 | 1 | 2 | 489 [255 ; 735] | 1361 [548 ; 1875] |
| 4-4 | 1 | 3 | 941 [709 ; 1287] | 2142 [1460 ; 3322] |
| 4-5 | 3 | 20 | 695 [576 ; 827] | 1452 [1113 ; 1945] |
| 4-6 | 1 | 3 | 898 [647 ; 1439] | 2253 [1578 ; 5981] |
| 5-5 | 1 | 3 | 194 [137 ; 322] | 276 [197 ; 641] |
| 6-8 | 8 | 165 | 106 [85 ; 135] | 286 [207 ; 480] |
